# Revisiting Subject–Object Asymmetry in the Production of Cantonese Relative Clauses: Evidence From Elicited Production in 3-Year-Olds

**DOI:** 10.3389/fpsyg.2021.679008

**Published:** 2021-12-23

**Authors:** Angel Chan, Stephen Matthews, Nicole Tse, Annie Lam, Franklin Chang, Evan Kidd

**Affiliations:** ^1^Department of Chinese and Bilingual Studies, The Hong Kong Polytechnic University, Kowloon, Hong Kong SAR, China; ^2^The Hong Kong Polytechnic University – Peking University Research Centre on Chinese Linguistics, Hong Kong, Hong Kong SAR, China; ^3^Research Centre for Language, Cognition, and Neuroscience, Department of Chinese and Bilingual Studies, The Hong Kong Polytechnic University, Hung Hom, Hong Kong SAR, China; ^4^Department of Linguistics, The University of Hong Kong, Pokfulam, Hong Kong SAR, China; ^5^Department of English Studies, Kobe City University of Foreign Studies, Kobe, Japan; ^6^Max Planck Institute for Psycholinguistics, Nijmegen, Netherlands; ^7^Research School of Psychology, The Australian National University, Canberra, ACT, Australia; ^8^ARC Centre of Excellence for the Dynamics of Language, Canberra, ACT, Australia

**Keywords:** Cantonese, child first language acquisition, elicited production, relative clauses, emergentism

## Abstract

Emergentist approaches to language acquisition identify a core role for language-specific experience and give primacy to other factors like function and domain-general learning mechanisms in syntactic development. This directly contrasts with a nativist structurally oriented approach, which predicts that grammatical development is guided by Universal Grammar and that structural factors constrain acquisition. Cantonese relative clauses (RCs) offer a good opportunity to test these perspectives because its typologically rare properties decouple the roles of frequency and complexity in subject- and object-RCs in a way not possible in European languages. Specifically, Cantonese object RCs of the classifier type are frequently attested in children’s linguistic experience and are isomorphic to frequent and early-acquired simple SVO transitive clauses, but according to formal grammatical analyses Cantonese subject RCs are computationally less demanding to process. Thus, the two opposing theories make different predictions: the emergentist approach predicts a specific preference for object RCs of the classifier type, whereas the structurally oriented approach predicts a subject advantage. In the current study we revisited this issue. Eighty-seven monolingual Cantonese children aged between 3;2 and 3;11 (*M*age: 3;6) participated in an elicited production task designed to elicit production of subject- and object- RCs. The children were very young and most of them produced only noun phrases when RCs were elicited. Those (nine children) who did produce RCs produced overwhelmingly more object RCs than subject RCs, even when animacy cues were controlled. The majority of object RCs produced were the frequent classifier-type RCs. The findings concur with our hypothesis from the emergentist perspectives that input frequency and formal and functional similarity to known structures guide acquisition.

## Introduction

Theories of language acquisition differ in how children’s grammatical competence should be characterized, the mechanisms proposed by which children can reach the adult-like grammar, and how the process and the nature of language acquisition proceeds. Emergentist approaches to language acquisition advocate that children are not born with adult-like syntactic knowledge, but that abstract categories and functionally driven knowledge of constructions emerge from the usage patterns in children’s linguistic experience and/or processing routines (e.g., Tomasello, 2003; O’Grady, 2005). Ontogenetically, children have to re-construct the grammatical dimension of language from the concrete linguistic expressions to which they are exposed with the aid of a set of cognitive, socio-cognitive and biological mechanisms. These mechanisms are domain-general, not specialized only for language learning, and involve interaction of multiple factors that are not inherently grammatical in nature, such as experience, cognition, processing, and function (O’Grady, 2011).

A prominent emergentist approach to language acquisition, the usage-based or “constructivist” approach (e.g., Lieven and Tomasello, 2008) adopts a constructional view of grammatical organization in cognitive linguistics (Fillmore et al., 1988; Goldberg, 1995; Croft, 2001) that aims at a unified representational account of all grammatical knowledge. Constructions are viewed as symbolic units, being integral pairings of form and meaning/function, and the notion of a construction is extended to cover linguistic structures of all levels of complexity (from morphological markers to lexical items to complex syntactic constructions) and schematicity. Linguistic competence is characterized in terms of the mastery of a structured inventory of meaningful linguistic constructions of a particular language (Langacker, 1987). Extending to language acquisition, what children eventually acquire is a network of constructions (see Diessel, 2020, this volume). In this network, constructions are related through specific links; and these links, which are non-derivational ways to capture the constructional relationships, are also part of the knowledge of the mental grammar.

On this theoretical perspective, the acquisition of constructions is potentially influenced by related (or neighboring) constructions, i.e., constructions with overlapping semantic and/or structural properties. One relevant hypothesis along these lines is the “construction conspiracy hypothesis,” proposed by Abbot-Smith and Behrens (2006), who propose that the acquisition of a new construction could be supported by the prior acquisition of simpler related constructions. In support of the hypothesis, they demonstrated that one German-speaking boy’s acquisition of the *sein*-passive was supported by his prior acquisition of the simpler *sein* copula construction (as a source construction), while this was not the case for the *werden*-passive. A similar phenomenon was described as “constructional grounding” in Johnson (1999); see also Israel et al. (2000). Moreover, some constructivist approaches have shown that form-based similarity can support the learning of complex constructions. For instance, Lewis and Elman (2001) and Reali and Christiansen (2005) suggested that complex syntax such as correct auxiliary fronting in interrogatives with RCs can be learnt by bootstrapping from simpler sentences present in the input. Others have argued that meaning-based similarity is critical for acquiring the appropriate rules. Fitz and Chang (2017) found that a connectionist model that learned to map between relative clauses (RCs) and multiple messages could not only acquire correct auxiliary fronting rules, but could also explain some of the errors that children make in acquisition when they incorrectly link meaning and form [e.g., double auxiliary errors in the question “*Is the boy who is watching Mickey Mouse is happy?” (Fitz and Chang, 2017, p. 236)].

Emergentism also embraces the natural variations in form-function mappings between languages, as languages differ in their ways of encoding particular functions (e.g., MacWhinney and Bates, 1989; Chang, 2009). Typological differences between languages can lead to cross-linguistic differences in the distributional regularities of form-function mappings, resulting in natural variations in the input properties of learners acquiring different languages. Since language structure emerges from aspects of language use, this approach identifies a core role for language-specific experience in syntactic development. Specifically, frequency assumes an explicit theoretical status in the emergentist approach (Ambridge et al., 2015). The human processor shows a general sensitivity to frequency that shapes the use and acquisition of language in explicit ways (O’Grady, 2011). This perspective therefore expects a clear influence/effect of frequency in the acquisition and processing of grammatical constructions.

Emergentist perspectives directly contrast with the nativist approach, which conceptualizes grammatical development as guided by Universal Grammar (UG). In UG approach to language acquisition, children’s hypothesis space is restricted by a set of innate language-specific principles and constraints that govern all human languages. This approach is also structurally oriented, as structural factors are primary determinants in affecting acquisition of grammar [as opposed to information peripheral to grammar, such as its frequency of usesuch as its frequency of use; see also works by Charles Yang (e.g. Li et al., 2021) which may be viewed as an exception]. They also have a radically different perspective to consider the theoretical status of constructions. Constructions are epiphenomena, generated by general syntactic principles and abstract features (Tomasello, 1998).

### Emergentist Versus Universal Grammar Structurally Oriented Perspectives: Acquisition of Relative Clauses

We next discuss how these two opposing theoretical perspectives conceptualize acquisition of RCs, focusing on the target constructions under current investigation. Working under an emergentist approach to language, O’Grady (2011) proposed a processing-based account for the acquisition of RCs, which is particularly relevant and useful in discussing the current study. Under this constraint-based approach to processing, there are multiple factors interacting to determine processing cost. He highlighted two factors that are particularly relevant to RCs: (i) prominence of the subject argument; and (ii) the cost of maintaining filler-gap dependencies.

The first factor is related to the functional notion of topicality. A RC functionally describes the referent of its head noun and there is a general subject prominence advantage in interpreting the “missing” argument in general: given that a clause’s subject is often the default topic, it is less effortful to parse a RC as being about its default topic (the subject) than to parse it as being about some other items (Keenan and Comrie, 1977; Kim and O’Grady, 2015). This factor therefore favors a general subject RC (SRC) over object RC (ORC) advantage across languages.

The second factor considers the linear length of the dependency relationship that holds between the modified nominal (the so-called “head noun” and the “filler”), and “the position at which it can be associated with the verb’s conceptual structure” (the so-called “gap,” O’Grady, 2011, p. 21), hence the so-called “filler-gap dependency.” Such a dependency places a burden on the processor to resolve the dependency relationship. As such, the longer the linear distance of the filler-gap dependency (when there are more discourse referents intervening between the filler and the gap), the more postponed the resolution of the dependency is, the more taxing it would be for working memory, and thereby the heavier load it is on the processor which is constrained in its processing capacity. In the case of English SRCs versus ORCs [see (1) and (2) below], the filler-gap dependency in SRCs can be resolved at a much lower cost to working memory than in ORCs, because there are fewer discourse referents intervening between the filler and the gap. This factor therefore favors a SRC over ORC advantage in a language like English.

**English subject RC**


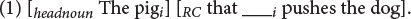


**English object RC**


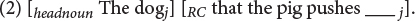


Regarding the role of related constructions in acquisition, there has also been research addressing the influence of related constructions on the acquisition of RCs in particular. The specific hypothesis is that the acquisition of RCs is facilitated if RCs bear (some) resemblance with main clauses. A precursor of this perspective dated back to a classic study by Bever (1970). More recent studies that have explicitly argued for the facilitating effect of main clauses on the acquisition of RCs in the framework of construction grammar include Diessel and Tomasello (2005); Diessel (2007), Brandt et al. (2008), Fitz et al. (2011), and McCauley and Christiansen (2019). In a language like English, SRCs (but not ORCs) will be facilitated as SRCs resemble SVO transitive main clauses.

**FIGURE 1:**
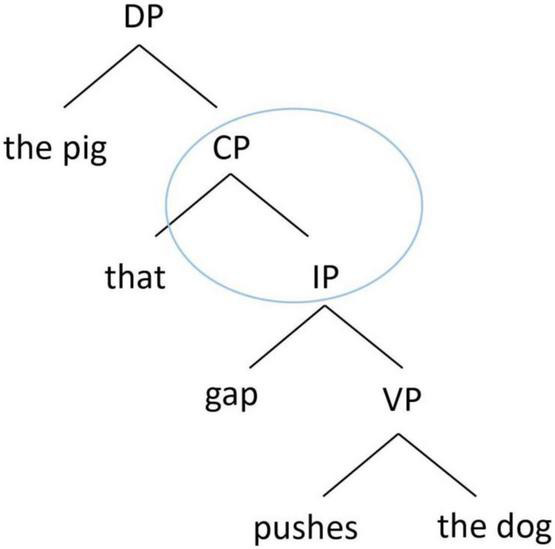
Hierarchical structure of an English subject RC.

**FIGURE 2:**
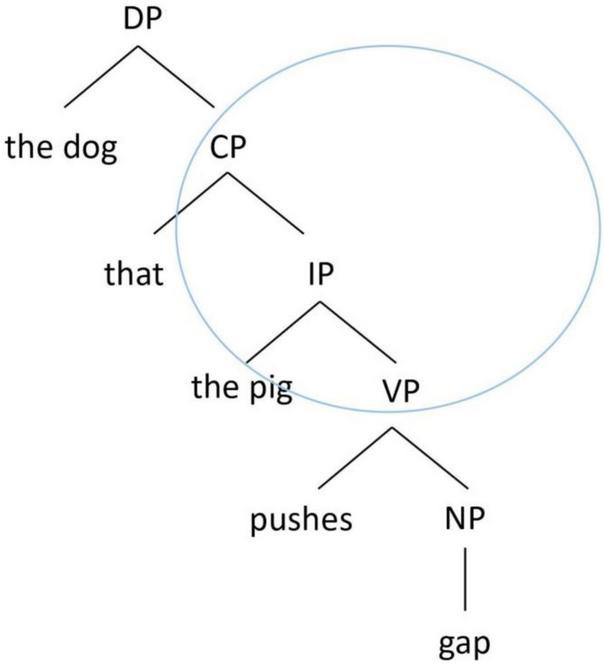
Hierarchical structure of an English object RC.

By contrast, the structurally oriented approach relies on hierarchical syntactic representations to consider the processing cost associated with the intervening elements between the filler and the gap when conceptualizing the acquisition and processing of RCs. We highlight two major types of structural factors that have been considered in the RC acquisition literature. The first type considers the structural distance between the filler and the gap, in terms of the depth of embedding of the gap in a hierarchical structure (e.g., O’Grady, 1997; Hawkins, 2004; Lin and Bever, 2006). There are various metrics in how the structural distance is computed, but the basic idea is that the deeper a gap is embedded in the hierarchical structure, the longer the structural distance it is, and the more difficult it is to process. Taking English RCs as an example, a SRC as in Figure 1 has a shorter hierarchical structural distance between the filler (“the pig”) and the gap than an ORC as in Figure 2. Therefore, in English, a SRC is easier to process than an ORC.

Another structural factor is structural intervention (Friedmann et al., 2009). A dependency is harder to process when there is a structural intervener, which violates Relativized Minimality (Rizzi, 1990, 2004), which places local constraints on dependencies in a sentence. In an English ORC as in Figure 2, the dependency between the head noun (“the dog”) and its gap site has to cross over the embedded subject of the RC (“the pig”). Since the embedded subject is identical in some formal features with the head noun (e.g., both are animate lexical NPs), the RC-internal subject becomes a structural intervener blocking the local relation between the head noun and its gap site, violating Relativized Minimality. By contrast, in an English SRC as in Figure 1, there is no structural intervener in the dependency between the head noun (“the pig”) and its gap site, and therefore its processing is computationally less demanding. As such, in English, a SRC is again easier to process than an ORC.

The structurally oriented approach therefore predicts that in a language like English, SRCs would be easier than ORCs to acquire/process, because of shorter structural distance and lack of structural intervention associated with SRCs. Note that emergentism and structurally oriented theories both make the same prediction for SRC over ORC advantage in acquisition in this case, despite a different underlying nature of difficulty for ORCs, and therefore one cannot test these two opposing theories in a language like English.

### Post-nominal Versus Pre-nominal Relative Clauses: Subject/Object Asymmetry in L1 Relative Clause Acquisition

Looking beyond English, there is a need to examine how these factors apply crosslinguistically and across diverse typological contexts [see Lehmann (1984) for a typological overview of RCs]. One case we study here is the rare combination of head-final pre-nominal RCs where RCs are placed before the head noun that they modify.

In a post-nominal RC language like English, the two factors prominence and distance appear to coalesce, acting in synergy to create a strong bias favoring SRCs over ORCs. Similarly, as mentioned, the structural factors considered in the structurally oriented theories would also favor SRCs over ORCs. These predictions align with the findings reported in the L1 acquisition literature. A large body of acquisition literature has demonstrated that in English and other European languages with head-initial RCs, SRCs are consistently easier to process/acquire than ORCs when animacy is controlled (e.g., English and German: Diessel and Tomasello, 2005; French: Labelle, 1990, 1996; Hebrew: Friedmann et al., 2009; Arnon, 2010; Italian: Adani, 2011; Contemori and Belletti, 2014).

However, when one considers the L1 RC acquisition literature on the issue of subject/object asymmetry in head-final post-nominal RC languages like Japanese, Korean, and Chinese, we see a much less consistent pattern of results across a growing body of acquisition studies. The mixed findings suggest either a lack of a robust SRC over ORC advantage, or even an opposite pattern of an ORC over SRC advantage.

In the L1 Japanese RC acquisition literature, studies have reported mixed findings that point to a lack of a robust subject over object advantage. For example, Harada et al. (1976) used an act-out task to test 98 Japanese-speaking children aged between 3;6 and 10;11 and found no effect of the gap position. Hakuta (1981) tested 12 preschool children aged 5;3 and 6;2 in an act-out task and found an object advantage. Based on analyzing the longitudinal naturalistic production data of five Japanese-speaking children aged from birth to 3;11, Ozeki and Shirai (2007) found no marked difference between SRCs and ORCs. In a more recent study, Suzuki (2011) constructed a picture description task to test L1 monolingual Japanese-speaking children aged between 5;1 and 6;8 and found no difference in the difficulty between SRCs and ORCs for the children who could use case markers for the comprehension of single-argument sentences. Most recently, Sasaki et al. (2021) tested Japanese-speaking children on their comprehension of RCs using a picture pointing task, and reported a subject over object advantage in their typically developing children.

A similar phenomenon pointing to a lack of a robust subject over object advantage happens in the L1 Korean RC acquisition literature too. For instance, Kim and O’Grady (2015) compared production of RCs in child English versus Korean and found SRC advantage in both groups. However, Yoo and Yim (2021) reported no SRC over ORC advantage in both online and offline comprehension in typically developing Korean-speaking children, using a self-paced reading task and a picture selection task, respectively.

In the L1 Mandarin RC acquisition literature, corpus studies of children’s spontaneous speech and adult input (Chen and Shirai, 2015; Liu, 2015) reported that ORCs were more frequent and emerged earlier than SRCs in both children’s speech and adult input. However, these early ORCs were also restricted in form and function (e.g., most of these ORCs were isolated noun phrases without a main clause, and typically modify inanimate head nouns), and therefore they may not demonstrate mastery of the construction. Experimental studies have yielded mixed findings, with some studies showing SRC over ORC advantage (Lee, 1992; Hsu et al., 2009), others showing ORC over SRC advantage (Ning and Liu, 2009), and some reporting no difference (Chang, 1984; Su, 2004; see Chan et al., 2011 for a review). However, many early studies had their methodological limitations, and more recent studies appear to show a more consistent subject over object advantage in comprehension (Hu et al., 2016b; Tsoi et al., 2019) and production (Hsu, 2014; Hu et al., 2016a).

This apparent subject over object advantage appears to be consistent with predictions from structurally oriented perspectives for Mandarin (Hu et al., 2016a,b). However, Mandarin has different SRC and ORC constructions, and these past experimental studies had only assessed RCs with the relative marker *de* introducing a bare head noun (termed DE-RCs in Yang et al., 2020), but not another productive RC type where the relative marker *de* introduces a head noun that is followed by the demonstrative *that* and classifier (CL) (termed DCL-RCs in Yang et al., 2020). See examples (3) to (6).

**Mandarin subject DE-RC:**


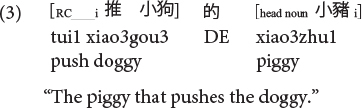


**Mandarin object DE-RC:**


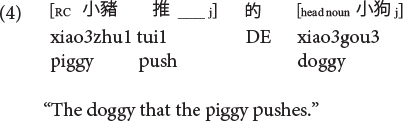


**Mandarin subject DCL-RC (CL: classifier):**


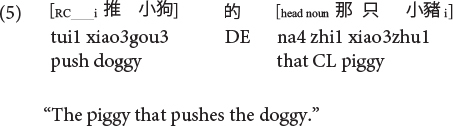


**Mandarin object DCL-RC:**


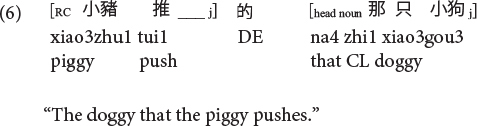


Comparing the acquisition and processing of DE-RCs versus DCL-RCs in Mandarin is theoretically illuminating, because these two RC types not only differ- and are reversed- in their distributional properties in the adult input: SRC-like structures are more frequent than ORC-like structures for the DE type; but ORC-like structures are more frequent than SRC-like structures for the DCL type. Our recent study (Yang et al., 2020) is the first examining the online comprehension of DE-RCs versus DCL-RCs in a group of Mandarin 4-year-olds using a within-subjects design, and reported that the children displayed subject advantage in DE-RCs (as in some previous studies), but the same children showed an object advantage in DCL-RCs. These findings cannot be readily explained by structurally oriented perspectives, but align with predictions from emergentist experienced-based accounts that expect developmental processing preferences being shaped by distributional frequencies in the learner’s experience.

Turning to Cantonese, the target language under current investigation, there has been no published corpus study of naturalistic speech reporting on the acquisition of Cantonese RCs in monolingual Cantonese-speaking children. Existing studies of child Chinese are based on naturalistic speech of bilingual Cantonese children and monolingual Mandarin children in Yip and Matthews (2007) and Chen and Shirai (2015) respectively, which reported that ORCs are attested earlier than SRCs. However, studies of naturalistic speech have two limitations: there could be more opportunities for using ORCs in these naturalistic samples; and the early ORCs attested are restricted in form and function, many of them being isolated noun phrases without a main clause and typically modify inanimate head nouns. As such, for our collective understanding it is more informative when equal opportunities are provided to elicit SRCs versus ORCs and when animacy cues are neutralized in experimental investigations.

There are experimental studies that controlled these two factors. Using a picture identification task and a picture description task, Lau (2016b) studied the RC comprehension and production of monolingual Cantonese children, aged 3;0–5;11 and 4;03–5;10 respectively, and reported that children showed better performance on SRCs than ORCs in her picture identification comprehension task and no overwhelming preference for either SRCs or ORCs in her picture description production task. A more recent study by Chan et al. (2018) examined the online comprehension of SRCs and ORCs in Cantonese 4-year-olds, and reported a weak object over subject advantage in the comprehension of classifier RCs [see examples (9) to (11) below], but a subject over object advantage in the comprehension of GE-RCs [see example (13) below]. Again, these findings challenge the structurally oriented approach to acquisition and processing which would predict a uniform subject over object advantage for Cantonese for both RC strategies, since the results suggest that comprehension is significantly guided by distributional frequency information in children’s linguistic experience.

### Mandarin Versus Cantonese Relative Clauses

In this section, we highlight below the similarities and differences between Mandarin and Cantonese, as a preface for elaborating on the specific predictions of emergentism versus structurally oriented theories for Cantonese RC acquisition in the next section. In discussing the similarities, we explain why the effects of distance and prominence would pull in opposite directions in both languages. In discussing the differences, we highlight how Cantonese, the target language under investigation, also differs from Mandarin.

#### Similarities

Sinitic languages like Mandarin and Cantonese are exceptional among SVO languages in placing the RC before the head noun (Keenan, 1985; Dryer, 2013). Given this configuration, it is the ORCs, not the SRCs, that have a shorter length of the filler-gap dependency. Compare (5) versus (6) repeated as (7) versus (8) in Mandarin and (9) versus (10) in Cantonese.

**Mandarin subject RC (CL: classifier):**


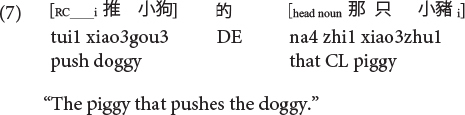


**Mandarin object RC:**


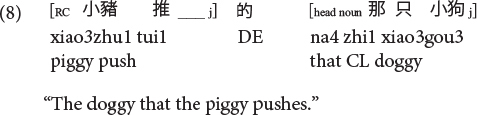


**Cantonese subject RC:**


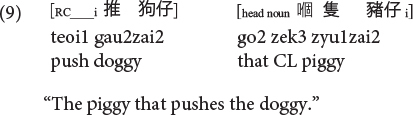


**Cantonese object RC:**


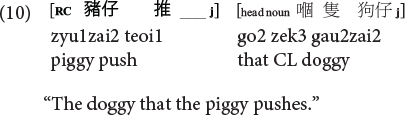


Moreover, it is also the ORCs not the SRCs that follow the canonical SVO word order, and resemble frequent and early-acquired simple SVO transitive clauses. Consider the hypothesis that the acquisition of RCs is facilitated if RCs bear resemblance with main clauses, ORCs (but not SRCs) would be facilitated from this emergentist constructivist perspective. Consequently, the distance factor and facilitation from simple main clauses would favor ORCs (not SRCs) in these two Chinese languages, exerting an opposite effect from the prominence factor which would favor SRCs (not ORCs) across languages in general.

#### Differences

On the other hand, Cantonese is unique among South East Asian languages according to the functions of classifiers. Unlike Mandarin, classifiers in Cantonese (and some other Southern Sinitic languages and Miao-Yao languages) have undergone grammaticalization with their functions extending from not only individualization and classification but also to referentialization and relationalization (Bisang, 1993; Matthews and Yip, 2001). Consequently, classifiers in Cantonese are multi-functional and can serve as a referential marker indicating specificity and a RC marker as an instance of relationalization in noun phrases. Table 1, adapted from Matthews and Yip (2001, Table 10.1), based on Bisang’s (1993) typology, nicely classifies these South East Asian languages according to the functions of classifiers.

**TABLE 1:** Functions of classifiers in South East Asian languages (adapted from Matthews and Yip, 2001, Table 10.1).

Type	Functions of classifiers	Languages
III	Individualization, classification, referentialization, and relationalization	Cantonese, Hmong, Weining Miao
II	Individualization, classification, and referentialization	Thai, Vietnamese
I	Individualization and classification	Cambodian, Mandarin

As such, Cantonese classifier ORCs not only resemble but are ***identical*** in surface form with SVO main clauses, because the classifier itself can serve as a RC marker in this language. Compare (10), repeated below as (11) and (12).

**Cantonese object classifier RC:**


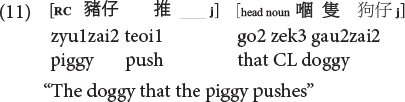


**Cantonese transitive SVO main clause:**


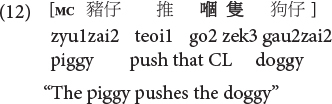


Sentences (9) to (11) are called classifier RCs (henceforth CL-RC) because the classifier serves as the relative marker. CL-RCs are frequently used in spoken Cantonese, especially in informal register, and in adult child-directed speech (Chan et al., 2018). Cantonese has two more formal relativization strategies that are similar to Mandarin RCs, where RCs are marked by the particle *ge3* [see (13), called GE-RCs here] or marked by both *ge3* and classifier [see (14), called hybrid GE-CL RCs here].

**Cantonese object RC of the *ge3* type (GE-RC) (PRT: particle):**


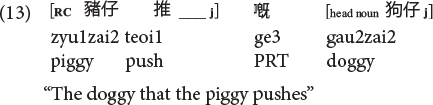


**Cantonese object RC of the hybrid type (hybrid GE-CL RC):**


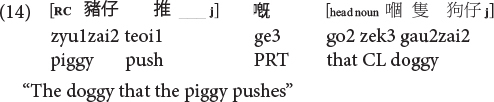


Note that this surface identity with SVO transitives is unique to Cantonese object classifier RCs, but not Mandarin ORCs. Mandarin ORCs as in (4), (6), and (8) only resemble but are not identical in surface form with SVO transitive main clauses, due to presence of the relative marker *de*. Regarding identical surface form, it is also natural to wonder whether there are prosodic differences between the two constructions. Lau (2016a) attempted to elicit native Cantonese adult speakers’ production of object classifier RCs and SVO transitive main clauses, which were identical in surface form. Interestingly, an acoustic analysis showed no prosodic differences between the two structures. While more in-depth investigations are needed for further research, there is thus far no empirical evidence suggesting that adult native speakers of Hong Kong Cantonese use prosody to disambiguate the surface identity in syntax between Cantonese object classifier RCs and transitive main clauses.

If this is so, Cantonese is unique in at least two more ways. First, given the surface ***identity*** and functional overlap with SVO transitive main clauses, the weight of facilitation effect from simple main clauses may be even stronger in Cantonese for its object CL RCs (compared to the case of Mandarin ORCs being differentiated by a relative marker *de*), in production. We highlight production here because the current study is a production study and the effect could be different in comprehension (see section “Discussion”). Second, the identity in surface form and the overlap (but not being identical) in function between object CL RCs and SVO transitive main clauses in Cantonese offers a good demonstration for the important role of function for disambiguation. This point is also consistent with a central orientation of emergentist usage-based linguistics: the importance of function as a crucial factor in finding and creating linguistic patterns, both historically and developmentally (Tomasello, 2003).

### Predictions for Cantonese Relative Clauses: Emergentism Versus Structurally Oriented Theories

As mentioned in the theoretical introduction, frequency has an important theoretical status in emergentism (Ambridge et al., 2015). Abbot-Smith and Behrens (2006), for instance, argued that “input frequency should be examined in relation to a network of related constructions, rather than in relation to a construction in isolation.” We therefore conducted a corpus study of adult-to-child directed speech from two monolingual Cantonese corpora that are available on the CHILDES database,^1^ namely CanCorp (Lee and Wong, 1998) and HKU-70 (Fletcher et al., 2000). These two corpora contained a total of 241 transcripts from 78 Cantonese speaking children (half female) aged between 1;07 and 5;6. We extracted all adult utterances containing classifier (CL) and *ge3* using the Computerized Language Analysis (CLAN) program (MacWhinney, 2000). Since CL and *ge3* are multi-functional in Cantonese, the extracted data were further manually disambiguated and coded.

Similar to Vasishth et al. (2013) and our previous work (Yang et al., 2020), we targeted utterances that are more general than genuine RCs, the so-called “RC-like” sequences. These sequences were RC-like because they have the same surface form as Cantonese SRCs [V-N-(*ge3*)-(D)CL-(N)] and ORCs [N-V-(*ge3*)-(D)CL-(N)], and we further restricted our current level of analyses to noun modifying constructions. As such, they include both conventional RCs (where a filler-gap dependency can be readily conceived) and gapless noun modifying clausal constructions [see (15) and (16)] which have the same surface form and share the discourse-functional properties with conventional RCs as noun-modifying constructions.

**Gapless noun-modifying constructions in Cantonese:**


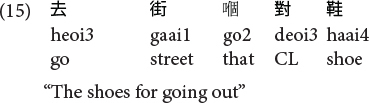



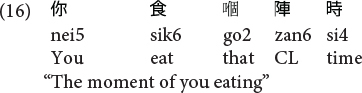


Table 2 lists the structural frequencies of SRC-like and ORC-like sequences for (D)CL, GE, and hybrid (*ge3* + CL) constructions, which map onto the three relativization strategies in Cantonese. Overall, (D)CL RC-like sequences were far more frequent than GE RC-like sequences, with hybrid RC-like sequences unattested (273 tokens versus 78 tokens versus 0 tokens), consistent with the fact that CL RCs are commonly used in colloquial speech, while the other two relativization strategies (GE and hybrid) are more commonly used in formal registers. Across both (D)CL and GE RC-like sequences, ORC-like sequences were noticeably more frequent than SRC-like sequences [1.5 times more frequent for (D)CL and 1.9 times more frequent for GE]. Note that the current level of analyses has not yet counted the SVO transitive constructions which share the same surface form with object CL RCs [N-V-(D)CL-(N)] and has functional overlap with object CL RCs at the semantic level of agent-patient relations (the current level counted only also the gapless noun modifying clausal constructions which are functionally closest to conventional RCs). If we were to go beyond this more conservative level of analysis adding also those frequently used SVO transitives, ORC-like sequences would be even far more frequent than SRC-like sequences, i.e., >1.5 times more frequent, for (D)CL [see also Chan et al. (2018) reporting that simple transitives which share surface identity with object CL RCs were twice as frequent as object CL RCs in their corpus study of Cantonese adult child-directed speech].

**TABLE 2:** Frequencies of (D) CL, GE, and Hybrid RC-like noun modifying constructions in Cantonese child-directed speech.

RC strategy	SRC-like	ORC-like
(D) CL	109	164
GE	27	51
Hybrid	0	0

*D, demonstrative; CL, classifier; GE, ge3.*

Specifically, one unique developmental prediction from the emergentist approach would be that ORCs, object CL-RC in particular, would be facilitated, because of its high structural frequencies in young children’s linguistic experience. Moreover, the distance factor would also favor ORCs over SRCs in general. Furthermore, on the basis of shared structural properties, and overlap in functional properties at the semantic level of agent-patient relations, object CL-RC as in (11) and transitive construction as in (12) could be conceptualized as related constructions in a construction network. Children can make use of the simpler and earlier acquired transitive construction (as a source or supporting construction) to bootstrap onto formulating an object classifier RC in production. Consider that emergentism views acquisition as a multi-factorial adaptive system with different factors interacting or even competing over the course of development, competition is a major theoretical theme (Bates and MacWhinney, 1987; Hawkins, 2007). In this regard, Cantonese RCs are intriguing in light of competing constraints in emergentism (MacWhinney, 2005, 2012) because, unlike in commonly studied languages like English, these factors of input frequency, distance, and support from related constructions that favor ORCs (and the CL type in particular) may conspire to override subject prominence in Cantonese.

**FIGURE 3:**
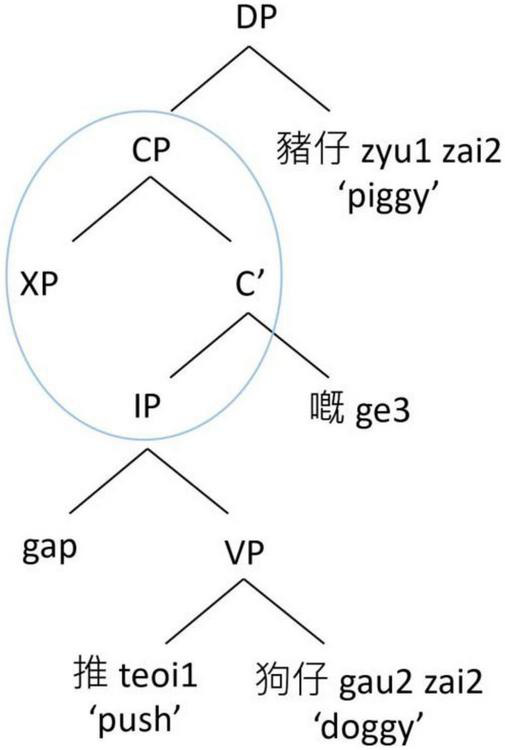
Hierarchical structure of a Cantonese subject RC.

**FIGURE 4:**
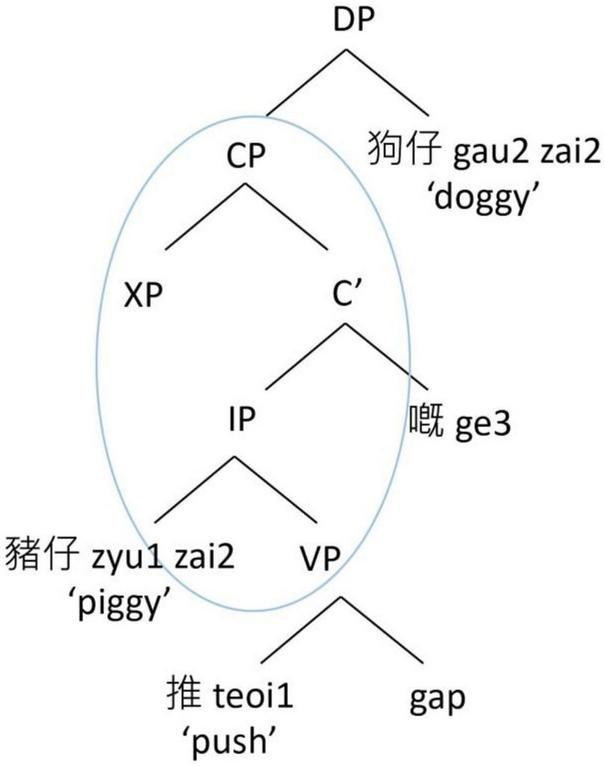
Hierarchical structure of a Cantonese object RC.

In contrast, the structurally oriented approach to RC acquisition (O’Grady, 1997; Friedmann et al., 2009; Hu et al., 2016a,b) considers that structural factors are primary determinants in affecting acquisition of grammar (as opposed to information peripheral to grammar, such as its frequency of use); and as such consider complexity based on formal complexity rather than experience. In Cantonese SRCs like Figure 3, the structural distance between the filler (the head noun *zyu1zai2* “the piggy”) and its gap site is shorter. There is also no structural intervener between the head noun and its gap site. On the other hand, in Cantonese ORCs like Figure 4, the structural distance between the filler (the head noun *gau2zai2* “the doggy”) and its gap site is longer. There is also a structural intervener (the embedded RC-internal subject *zyu1zai2* “the piggy”) between the head noun and its gap site. This approach therefore would predict a subject advantage also for Cantonese RCs, since ORCs are considered computationally more demanding to process.

### Current Study

As an extension to our previous works on RC comprehension in child Cantonese (Kidd et al., 2015; Chan et al., 2017, 2018), we extend our experimental work to production in the current study. Unlike previous acquisition studies assessing older children (e.g., Lau, 2016b), we target a group of younger children aged 3 years, aiming to capture how they attempt to produce RCs at an early stage of acquisition. Specifically, we test the developmental predictions from the emergentist perspectives and the structurally oriented approach: the former predict a specific preference for object CL-RCs, but the latter predicts a subject advantage.

## Materials and Methods

### Participants

Eighty-seven typically developing monolingual Cantonese-speaking children aged between 3;1 and 3;11 (33 male and 54 female) were recruited from six kindergartens in Hong Kong to participate in this study. Under the trilingualism and biliteracy language policy in Hong Kong, these children are considered predominantly monolingual in Cantonese, because they have been exposed to Cantonese as their first, family and community language from birth, and have been studying in a local school using Cantonese as the medium of instruction, without receiving regular, intensive and extensive exposure to other languages at home and outside of home. Exclusion criteria include children having a previous clinical diagnosis of language impairments or other developmental disorders, children having atypical language milestones of onset of first words and word combinations, and children whose parents have expressed concerns over their child’s development in language, hearing, or other areas of cognition. Parental questionnaires were collected to ensure that the children tested did not meet these exclusion criteria.

### Materials

Each participant received 4 practice trials (2 SRCs and 2 ORCs) and then 16 experimental trials (8 SRCs and 8 ORCs). See Supplementary Appendix A. All RC trials contain nouns that feature animate entities using common animal names, so that animacy cues were neutralized, and all verbs were transitive activity verbs. The test consisted of two different scripts. Each script contained 16 experimental trials, which included 8 SRCs and 8 ORCs in randomly order. Children were randomized assigned to one of the two scripts.

### Procedure

All children were tested individually by two female native Cantonese-speaking experimenters in a quiet room in their school. Each child took part in a task modeled after Crain et al. (1990) and Courtney (2011) to elicit production of SRCs and ORCs in a within-participants design. Crucially, each child and an experimenter (ExpA) observed as another experimenter (ExpB) manipulated two animal toy figures of the same type performing different actions. ExpA was then blindfolded and the child had to help ExpA identify the figure which ExpB pointed at using verbal reference.

The task runs as follows. In each trial, ExpB would be responsible for placing four animal toy figures in four pre-specified locations on the table, with the two animals of the same type (one target and one distractor) being placed diagonally, horizontally or vertically in different trials, and a different animal type (the related) being the animal which the target would interact with, and another different animal type (the unrelated) being the animal which the distractor would interact with and with a different action. ExpB introduced the task expectation to the child by saying the following (pointing cues are stated in parenthesis): “*Now I am going to play a game with you. You have a task, and your duty is to help this lady (point to ExpA) find an animal. Later you will see some animals (point to and name each of the 4 animals on table), and they will do different actions. Then, this lady (point to ExpA again) will wear a blindfold, and then I will point to one of the animals, for example this (point to one of the animal figures that are two tokens of the same type). This lady has her eyes covered so she cannot see, but she can still listen (point to ears), therefore you have to tell her which animal I am pointing to, so she can pick it up and give it to me.*”.

ExpB then reminded the child again by saying “*Remember to speak clearly. Do not only use your finger to point, or do not only say ‘this one’ or ‘that one,’ or do not only label the animal name. Because this lady has her eyes covered so she cannot see. You have to pay attention, remember what the animals are going to do, and then you can speak in a full sentence, to help this lady find the animal. Now let me show you how to play this game.*”

ExpB then started the practice trials. The child and ExpA then watched as ExpB manipulated the animal figures by performing different actions, presenting two background scenes (e.g., acting out one pig pushing the dog, and then the other pig tickling the monkey in the SRC condition; *or* acting out the cat chasing the duck and then the frog feeding the other duck in the ORC condition). While acting out each background scene, ExpB would describe the action by saying, e.g., *Look! This one pushes. The other one tickles*, so the child heard all the animal names and verbs needed for formulating a RC. After this, ExpA put on a blindfold, and ExpB pointed to one of the animal toy figures (e.g., the pig that tickled the dog), asking the child to help ExpA identify the target animal by verbally describing which figure she was pointing to, upon ExpB prompting *“Which one am I pointing to?”* The two background scenes created a felicitous discourse context for the use of a restrictive RC to modify and restrict the referent from a set. The order of mentioning the target referent in the background scenes was counterbalanced across trials, with half mentioned in the first background scene, and half in the second.

During the first four practice trials, ExpB would demonstrate to the child the production of the target RC responses, for concrete demonstration of the task expectation. In the first two practice trials (one SRC and one ORC), ExpB only expected the child to listen to the two RC models. In the last two practice trials (one SRC and one ORC), ExpB would ask the child to imitate her two RC models, to increase the child’s awareness that the blindfolded ExpA had to rely on the child’s verbal output to identify the target figure. The four RC models spoken by ExpB used the hybrid GE-CL RC type [see e.g., (14)], with a simple copula main clause, i.e., in the form of “It’s [RC] head noun.” This RC strategy was chosen as it has the merits of being able to clearly present the structure as an RC introducing the head noun with an explicit RC marker *ge3* (so no structural ambiguity) while still containing a classifier before the head noun which is commonly used in child-directed speech (although this *ge3*-classifier double marking is not necessary for grammaticality and is not frequently used in Cantonese child-directed naturalistic speech; see Chan et al., 2018). As long as the child showed compliance to attempt imitating the two RC models, the experimenters would proceed to the test trials, regardless of the child’s accuracy in imitation. After the child’s verbal response, ExpA removed her blindfold and identified the figure based on the child’s verbal description. No modeling of target RC responses was provided in the experimental trials. The first response produced by each child was recorded and then transcribed by the experimenters.

### Data Coding

The first response produced by each child was scored according to its production accuracy. One mark was given to each correct response and zero mark given for a non-target response. Correct response refers to production of an RC that matched the type of RC that the condition was designed to elicit (SRC or ORC), not restricting the use of the relativization strategy. Marks would not be deducted for minor lexical substitutions as long as the target RC structure was produced. The third and the fourth authors coded all the children’s responses. A research assistant coded 20% of the data (18 out of 87 children, 20.7%) for inter-rater reliability. Inter-rater reliability was close to 100% agreement.

## Results

The R package lme4 (Bates and Maechler, 2010) in R (version 3.5.1, R Core Team, 2018) was used to fit Generalized Linear Mixed Models (GLMM; Baayen et al., 2008; Jaeger, 2008). The fixed effect was RC type (SRC versus ORC; mean-centered), and the random effects for participants and items. The main effect of RC type (χ^2^ = 17.63, *df* = 1, *p* < 0.001) significantly improved the model, showing that children produced significantly more ORCs than SRCs (20 versus 3 tokens, β = 2.337, *z* = 3.496, *p* < 0.001).

At an early age of 3-year-olds, only a small number of children were able to produce a target RC (10.3%, 9 out of 87 children). This is expected since this study aimed to capture the emerging competence to formulate an RC among younger children acquiring Cantonese as a first language. Among these nine children who could produce a target RC, the three children who each produced a SRC token (TP_06, TP_02, and TP_03) were also able to produce 1–3 ORC tokens at the individual level (see Table 3). Moreover, Table 4 tabulates the relativization strategy used in the 23 tokens of target RCs produced, showing that 60% of the tokens were CL-RCs (14 out of 23 tokens), followed by GE-RCs (7 out of 23 tokens), and last by hybrid RCs marked by both *ge3* and a classifier (2 out of 23 tokens). Supplementary Appendix B lists all the target RCs produced by the children.

**TABLE 3:** Individual performance of participants who could produce a target RC.

Participants who produced target RC(s)	Number of target SRC produced	Number of target ORC produced	Total number of target RCs produced
1 (DH_04)	0	2	2
2 (DH_05)	0	4	4
3 (DH_08)	0	1	1
4 (TKW_18)	0	3	3
5 (TP_06)	1	3	4
6 (TP_08)	0	3	3
7 (TP_10)	0	1	1
8 (TC_02)	1	1	2
9 (TC_03)	1	2	3
		Total number of target RCs:	23

**TABLE 4:** Distribution of target RCs produced across relativization strategies and RC types.

RC strategy	SRC	ORC
(D) CL	2	12
GE	1	6
Hybrid	0	2

A further remark regarding the coding of object classifier RCs is warranted. As mentioned in the introduction section, object classifier RCs in Cantonese are unique because they share surface identity with simple SVO transitive constructions, unlike the other two relativization strategies which have a *ge3* particle as relative marker. One might therefore query whether these tokens of object CL-RCs should be coded as ORCs or as simple SVO transitive constructions. We decided to code these tokens as ORCs on the grounds that each of these tokens was not only correct in form but also expressed a referential (not declarative) function in the discourse context, which matches functionally with an RC (noun-modifying) construction rather than a simple SVO transitive construction. Moreover, as the error analyses below show, a majority of the non-target responses in both SRC and ORC conditions were single noun phrases referring to the target referent (64.1% in SRC condition; 70.6% in ORC condition), providing consistent illustrative evidence that these children displayed understanding of the task expectation that their verbal description should be a noun-referring expression in this referential task. Another source of evidence is that while there is surface identity between the SVO transitive and the CL-ORC in Cantonese, the CL is not obligatory in the SVO transitive, and a lot more often these children produced non-target SVO clauses without a CL introducing the object in the ORC condition (see description of error types below). Thus, since the children used SVO and SV-CL-O, their choice to use the CL likely reflects that they were using the CL to highlight the object NP as the referent in this small set of responses [note also in most of these responses, CL was used together with the distal demonstrative *go2* 嗰 (that) which is typical in Cantonese CL-RCs, although *go2* is not obligatory for CL-RCs, see Supplementary Appendix B].

The distributional frequency of the error types was next examined. Tables 5, 6 show each error type with its proportion and frequency of occurrence in the SRC and ORC conditions respectively. The most frequent error type across both conditions was single noun phrase (64.1%, 444/693 in SRC condition; 70.6%, 477/676 in ORC condition). The second most frequent error type across both conditions was ungrammatical, irrelevant, or uninterpretable responses (21.1%, 146/693 in SRC condition; 19.4%, 131/676 in ORC condition). Also in both conditions, utterances in SVO surface form ranked third and was the most frequent error type among all complete and well-formed clausal level non-target responses (9.2%, 64/693 in SRC condition; 7.0%, 47/676 in ORC condition). These responses were coded as non-target because there was no *ge3* marker nor classifier as a relative marker before the second NP, and therefore could not be considered as a grammatical ORC in Cantonese in terms of the target language grammar based on their surface forms. Supplementary Appendices C, D list the illustrative examples of each error type in the SRC and ORC conditions, respectively.

**TABLE 5:** Distribution of error types in the SRC condition.

Error types	Number of occurrence	Proportion of occurrence
NP only	444	64.1
Ungrammatical/irrelevant/uninterpretable	146	21.1
(It is) SVO	64	9.2
SV	15	2.2
Conversion error to ORC	12	1.7
VO	6	0.9
SRC with resumptive NP	3	0.4
Serial verb construction	3	0.4
	693	100

**TABLE 6:** Distribution of error types in the ORC condition.

Error types	Number of occurrence	Proportion of occurrence
NP only	477	70.6
Ungrammatical/irrelevant/uninterpretable	131	19.4
(It is) SVO	47	7.0
SV	13	1.9
SVO and with agent-patient role reversal errors	4	0.6
ORC with resumptive pronoun	2	0.3
VO	1	0.1
Serial verb construction	1	0.1
	676	100

## Discussion

The current study reports the first experimental production study of Cantonese 3-year-olds, aiming to capture younger children’s emerging competence in producing RCs at an early stage of acquisition. Using an elicited production task and a within-participants design, we examined the relative ease of producing SRCs and ORCs when children were given equal opportunities to produce SRCs versus ORCs in a supportive discourse context without the aid of animacy contrast cues. This is an area for which the two theories make opposing predictions. Emergentist approaches predict a specific preference for the ORC of the classifier type. Structurally oriented perspectives predict SRC over ORC preference.

The findings showed that these 3-year-olds produced ORCs at a significantly higher rate than SRCs, even when animacy cues were controlled in an experimental context. The object over subject preference found in the current study did not support the prediction of structurally oriented approach, where ORCs are considered computationally more demanding to process than SRCs in Chinese, because ORCs in Chinese involve structural intervention violating relativized minimality, while SRCs do not (Hu et al., 2016a,b). This ORC preference in young children’s elicited production, therefore, cannot be driven by differences in formal complexity.

Specifically, the current findings also showed that the majority of ORCs produced were CL-RCs, and this specific pattern of findings are most compatible with emergentist perspectives which expect a clear effect of frequency in the acquisition of RCs. Recall our corpus findings indicate a high structural frequency of S-V-CL-O in children’s input, which arise from higher frequency of use of ORC-like than SRC-like noun modifying constructions, much higher frequency of CL-RCs than GE-RCs and hybrid GE-CL RCs, and much higher frequency of SVO transitive constructions that share surface identity with object CL-RCs than RCs in general [see section “Predictions for Cantonese Relative Clauses: Emergentism Versus Structurally Oriented Theories” for the corpus findings and Chan et al. (2018)]. Here we find supportive evidence that the mechanism driving acquisition is frequency-sensitive, consistent with the emergentist assumptions [see Chan et al. (2018) for similar arguments]. Structurally oriented perspectives that are based on formal syntactic theory and complexity do not readily explain the frequency effects observed in this elicited production experiment.

We next discuss how the current findings relate to the two factors that are particularly relevant to RCs in O’Grady’s (2011) processing-based account for the acquisition of RCs: (i) prominence of the subject argument; and (ii) the cost of maintaining filler-gap dependencies. The current findings suggest that higher structural frequencies in experience and lower cost of maintaining filler-gap dependencies that are associated with Cantonese ORCs (and the classifier type in particular) can override subject prominence in this case, when we are considering production in very young children as young as 3-year-olds.

On the surface, this suggestion would appear to differ from O’Grady’s (2011) speculation for Mandarin that prominence might have a stronger effect in production than in comprehension in Mandarin Chinese, when he was referring to the subject advantage reported in Hsu et al. (2009) for their adult and older child participants (mean age 4:8) in production, but the object advantage reported in the adult comprehension study by Gibson and Wu (2013). These discrepancies appear to be age-related, which may be consistent with a role for working memory: it is possible that frequencies in a learner’s experience and the cost of maintaining filler-gap dependencies may have a stronger effect in very young children especially when their working memory is more constrained in its capacity than older children and adults. On the other hand, for older children and adults who are relatively less constrained in its working memory capacity, it is possible that subject prominence could override distance and experience effects, as O’Grady (2011) speculated.

These speculations are related to our observations that these 3-year-olds tested in the current study also showed a significant object over subject advantage in another experimental RC production task using sentence repetition, but in another study of ours (Lai et al., in prep) testing two older groups of Cantonese-speaking typically developing children using sentence repetition, the older group (4;7–7;6) showed neither subject nor object advantage and the much older group (6;6–9;7) showed even a subject over object advantage in their RC production. This observation is also consistent with Hsu (2014) reporting that Mandarin 5-year-olds, but not the 3- and 4-year-olds, exhibited a clear SRC advantage in a RC sentence repetition experiment, and suggested that developmental and processing constraints such as working memory capacity associated with age may affect children’s patterns of subject/object asymmetry. However, age-related changes in subject/object asymmetry could be due to working memory and/or experience, and it is often difficult to divorce effects that look like working memory from experience. Future research could explicitly test the predictive validity of working memory in accounting for the variations observed in children acquiring Cantonese RCs.

Theoretically, these ideas are compatible with the emergentist perspectives because it is possible that the effects of multiple factors could vary in strength in development, giving rise to variation in SRC/ORC preferences as children grow older. Future research, ideally using a longitudinal design, could further examine how the pattern of subject/object preference changes over time as children grow in development at different ages. Structurally oriented accounts of acquisition, however, do not readily explain the shifts in subject/object asymmetry during the course of a child’s development.

We further discuss the role of related constructions in the acquisition of RCs, a point of emphasis for emergentist perspectives in a constructivist approach to language acquisition. As mentioned in the introduction, one unique characteristic of the predominant type of early ORCs produced, specifically object CL-RCs, is that they share surface identity with frequent and early-acquired SVO transitive clauses, a distinctive characteristic of Cantonese grammar which differs from Mandarin and other languages. Moreover, recall the non-RC patterns in children’s responses showing children’s tendency to use SVO structures: utterances in SVO surface form was the most frequent type of complete and well-formed clausal level non-target response in the ORC condition. We hypothesized that it is possible for young children to use SVO transitive as a source construction to bootstrap onto formulating object CL-RCs in production, and as such facilitates the production of ORCs in young children. Future research could make use of a dense database (Abbot-Smith and Behrens, 2006) to pursue this constructivist idea further by tracking in greater detail the possible relationship between the SVO transitive construction (as a source construction) and the more complex object classifier RC construction in early grammar.

While the specific mechanisms of emergence of object CL-RCs from transitives are unclear at this point, children have to recognize that SVO transitives and object CL-RCs are overlapping but distinct constructions: they are identical in surface form and overlap in the agent-patient configuration at the semantic-level; but are different in discourse-functional properties because SVO transitive is declarative in function, expressing a causative event, while object CL-RCs is referential in function as a noun-modifying construction. Moreover, one crucial difference between these two constructions is that the classifier in the object CL-RCs, compared to the classifier (if present) that introduces the second noun object in the transitive construction, functions not only as a marker of individualization and classification but also as a marker of referentialization and relationalization (Bisang, 1993; Matthews and Yip, 2001). A further typological characteristic of Cantonese RCs is that conventional RCs in Cantonese and certain Asian languages have been reclassified as a subset of noun-modifying constructions in the target language based on their overlaps in form and function (Comrie, 1996, 1998; Matthews and Yip, 2016, 2017). In this regard, the classifier also functions as a relational marker in other noun-modifying constructions, including not only the conventional RCs, but also gapless noun-modifying constructions, attributive constructions, and possessive constructions that are frequently used in adult child-directed speech.

**FIGURE 5:**
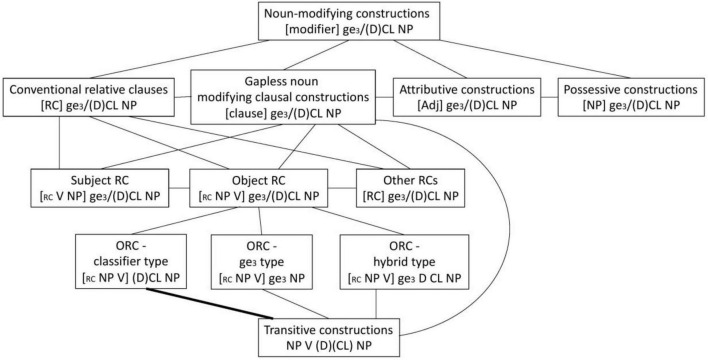
Relative clauses in a network of related constructions in Cantonese.

Given the above, Cantonese object CL-RCs can be conceived as connected to transitive SVO constructions and other noun modifying constructions that use classifier as a nominal particle in a network of constructions which may be conceptualised as in Figure 5.

How these constructional relationships emerge and how the more complex object CL-RCs emerge from simpler SVO transitives (and possibly from exemplars of other noun modifying constructions too) would likely involve processes such as analogy and categorization on the basis of both form and function in generalizing and abstracting schemas out of exemplars, functionally driven distributional analysis in detecting the functional similarity and contrast between congruent and competing forms, and extension and modification of the SVO transitive construction.

Moreover, we further hypothesized that the surface identity between object CL-RCs and SVO transitive construction could lead to facilitation in formulating object CL-RCs in production but errors in interpreting object CL-RCs in comprehension due to competing analyses as a result of structural ambiguity. Specifically, children might erroneously interpret the subject of the RC as the head noun in comprehension [i.e., assigning “piggy” instead of “doggy” as the head noun in (11)], due to competition with a SVO transitive interpretation. As in our previous works, we argue that the acquisition of Chinese RCs bears richly on the theoretical themes of competition and variation (Chan et al., 2011, 2017, 2018; Kidd et al., 2015). Our next follow-up paper aims to report on how the competing constraints affect production versus comprehension of RCs in the same 3-year-olds, testing this hypothesis further in a within-participants design.

Before moving to the conclusive remarks, we would like to further clarify that our current findings cannot be fully accounted for by simply attributing to Cantonese ORCs being similar to canonical SVO sentences in the target language. While similarity of ORCs to canonical SVO sentences is certainly relevant here (e.g., this could lead to higher structural frequencies experienced by children in their adult input), it is more than that. For example, similarity of ORCs to canonical SVO sentences alone could not account for the specific phenomenon that children preferred using ORCs of the classifier type [but not the other relativization strategies (GE and hybrid)] in their elicited production in this experimental study, because ORCs of the three relativization strategies are supposed to be all similar to canonical SVO sentences. Similarly, in two of our earlier experimental studies by Chan et al. (2018) and Yang et al. (2020) testing L1 Cantonese- and Mandarin-speaking children, respectively, the findings also could not be simply accounted for by similarity of ORCs to canonical SVO sentences.

Specifically, in Chan et al. (2018) and Yang et al. (2020), we documented that children showed different subject/object symmetry patterns between RC construction types when their online comprehension of SRCs versus ORCs was assessed: Cantonese-speaking children showed object over subject advantage in the CL type but subject over object advantage in the GE type; while Mandarin-speaking children showed object over subject advantage in the DCL type but subject over object advantage in the DE type. These findings could not be accounted for simply by referring to similarity of ORCs to canonical SVO sentences, because this factor would instead predict a uniform object over subject advantage across RC construction types, which was not the phenomena attested. Rather, we argued that the variations in subject/object asymmetry observed between RC construction types align with variations in the distributional properties of the children’s experience with these two construction types.

## Conclusion

This article reports the first experimental study of RC production that assessed Cantonese-speaking children as young as 3-year-olds, the youngest that have been tested in an experimental setting. We tested as many as 87 3-year-olds, and each child was given the opportunity to produce 16 RCs (8 SRC and 8 ORC). Out of these 1392 opportunities to produce an RC, most answers were simple NPs, and there were only 23 target RCs produced, capturing young children’s emerging ability to formulate RCs in production at such an early age. We reported a tendency for those children who did produce a target RC, to use ORCs more often than SRCs, displaying a object over subject preference in the elicited production experiment. They also displayed a selective preference toward CL-RCs over the other two RC strategies, where CL-RCs are more frequently encountered in children’s experience and object CL-RCs share surface identity with frequent and earlier acquired SVO transitives. These results challenge the structurally oriented approach that considers structural distance or structural intervention as the primary factor affecting processing cost, which predicts a subject over object preference in Chinese. Children’s early preference for object CL-RCs in elicited production aligns with our hypothesis from the emergentist perspectives that input frequency, distance, and support from related known constructions which favor object CL-RCs act in synergy to override subject prominence in early developmental Cantonese.^2^ This article demonstrates how language-specific properties affect the interaction of these factors in Cantonese, and how this in turn shapes developmental preferences in terms of the ease of producing SRCs and ORCs in early acquisition. Cantonese, being one of the best-known Sinitic languages in addition to Mandarin Chinese, offers a good opportunity to test the opposing predictions from emergentism versus structurally oriented perspectives in the acquisition of SRCs versus ORCs. In addition, given the multi-functionality of Cantonese classifiers that resemble the neighboring Southern Sinitic languages and Miao-Yao languages more than Mandarin Chinese, Cantonese offers a unique opportunity to discuss the role of function in the acquisition of RCs and its related constructions, where a functionalist approach to language is a major feature of emergentism.

## Data Availability Statement

The original contributions presented in the study are included in the article/Supplementary Material, further inquiries can be directed to the corresponding author.

## Ethics Statement

The studies involving human participants were reviewed and approved by the Human Subjects Ethics Sub-committee at the Hong Kong Polytechnic University (reference number: HSEARS20161230004). Written informed consent to participate in this study was provided by the participants’ legal guardian/next of kin.

## Author Contributions

AC designed the experiment and interpreted the data in consultation with the other co-authors. AC recruited the participants, supervised native-speaker experimenters and research personnel in data collection, coding, reliability checks and analyses, and wrote a first draft of the manuscript. AC, EK, FC, and SM worked on refining and revising the text. All authors approved the final version.

## Conflict of Interest

The authors declare that the research was conducted in the absence of any commercial or financial relationships that could be construed as a potential conflict of interest.

## Publisher’s Note

All claims expressed in this article are solely those of the authors and do not necessarily represent those of their affiliated organizations, or those of the publisher, the editors and the reviewers. Any product that may be evaluated in this article, or claim that may be made by its manufacturer, is not guaranteed or endorsed by the publisher.

## Footnotes

1
childes.talkbank.org

2 Despite the linear distance hypothesis by Gibson (2000) having been used in the RC acquisition/processing literature, it is not included in the major conceptual framing of this study. This is because this hypothesis is not an acquisition theory and is more relevant to comprehension data; but this study examined production. Moreover, even if one were to consider the linear distance hypothesis, it would predict a uniform ORC preference in prenominal RC languages like Cantonese, but would not predict a specific preference for ORC of the classifier type in Cantonese.
