# Supplementary material for: Revisiting Subject–Object Asymmetry in the Production of Cantonese Relative Clauses: Evidence From Elicited Production in 3-Year-Olds
Source: Front Psychol. 2021 Dec 23;12:679008. doi: 10.3389/fpsyg.2021.679008 (PMC8732946; doi:10.3389/fpsyg.2021.679008)
Supplement: Supplementary file 1 [file Data_Sheet_1.doc]

**Appendix A**

**Practice trials** [Subject relative clauses (2 items), Object relative clauses (2 items)]

P1: Subject relative clause

追 (嗰 隻) 牛牛 嘅 嗰 隻 熊貓

chase (that CL) cow ge3 that CL panda

The panda that chased the cow

P2: Object relative clause

(嗰 隻) 熊人 摸 嘅 嗰 隻 長頸鹿

(that CL) bear touch ge3 that CL giraffe

The giraffe that the bear touched

P3: Subject relative clause

嚇 (嗰 隻) 青蛙 嘅 嗰 隻 貓仔

scare (that CL) frog ge3 that CL cat

The cat that scared the frog

P4: Object relative clause

(嗰 隻) 狗仔 推 嘅 嗰 隻 馬仔

(that CL) dog push ge3 that CL horse

The horse that the dog pushed

**Experimental trials**

Target subject relative clauses to be elicited (8 items)

1. zit1 (嗰 隻) 羊仔 (嘅 / 嗰 隻 ) 豬仔

Tickle (that CL) sheep (ge3 / that CL ) pig

The pig that tickled the sheep

1. 追 (嗰 隻) 牛牛 (嘅 / 嗰 隻 ) 獅子

Chase (that CL) cow (ge3/ that CL ) lion

The lion that chased the cow

1. 咬 (嗰 隻) 馬仔 (嘅 / 嗰 隻 ) 熊貓

Bite (that CL) horse (ge3 / that CL ) panda

The panda that bit the horse

1. 踢 (嗰 隻) 大象 (嘅 / 嗰 隻 ) 長頸鹿

Kick (that CL) elephant (ge3 / that CL ) giraffe

The giraffe that kicked the elephant

1. 捉 (嗰 隻) 老虎 (嘅 / 嗰 隻 ) 熊人

Catch (that CL) tiger (ge3 / that CL ) bear

The bear that caught the tiger

1. 餵 (嗰 隻) 豬仔 (嘅 / 嗰 隻 ) 馬騮

Feed (that CL) pig (ge3 / that CL ) monkey

The monkey that fed the pig

1. 錫 (嗰 隻) 雞仔 (嘅 / 嗰 隻 ) 老鼠

Kiss (that CL) chicken (ge3 / that CL ) mouse

The mouse that kissed the chicken

1. 撞 (嗰 隻) 斑馬 (嘅 / 嗰 隻 ) 大象

Bump (that CL) zebra (ge3 / that CL ) elephant

The elephant that bumped the zebra

Target object relative clauses to be elicited (8 items)

1. (嗰 隻) 兔仔 抹 (嘅 / 嗰隻) 羊仔

(That CL) rabbit wipe (GE / that CL) sheep

The sheep that the rabbit wiped

1. (嗰 隻) 馬仔 踩 (嘅 / 嗰隻) 老虎

(That CL) horse step on (GE / that CL) tiger

The tiger that the horse stepped on

1. (嗰 隻) 貓仔 推 (嘅 / 嗰 隻) 鴨仔

(That CL) cat push (GE / that CL) duck

The duck that the cat pushed

1. (嗰 隻) 豬仔 嚇 (嘅 / 嗰 隻) 狗仔

(That CL) pig scare (GE / that CL) dog

The dog that the pig scared

1. (嗰 隻) 熊貓 舐 (嘅 / 嗰 隻) 斑馬

(That CL) panda lick (GE / that CL) zebra

The zebra that the panda licked

1. (嗰 隻) 雞仔 zit1 (嘅 / 嗰 隻) 白兔

(That CL) chicken tickle (GE / that CL) rabbit

The rabbit that the chicken tickled

1. (嗰 隻) 獅子 打 (嘅 / 嗰 隻) 牛牛

(That CL) lion hit (GE / that CL) cow

The cow that the lion hit

1. (嗰 隻) 馬騮 ong2 (嘅 / 嗰 隻) 貓仔

(That CL) monkey push (GE / that CL) cat

The cat that the monkey pushed

**Appendix B**

**Target RCs produced by the children**

**SRC**

1. 係 頭先 zit1 羊 嗰 隻 豬仔

Is just-now tickle sheep that CL piggy

It’s the piggy that just tickled the sheep

2. 係 踢 大象 嗰 隻 長頸鹿
 Is kick elephant that CL giraffe

It’s the giraffe that kicked the elephant

3. 打 大象 嘅 長頸鹿

Hit elephant ge3 giraffe

The giraffe that hit the elephant

**ORC**

1.豬仔 嚇 嘅 嗰 隻 狗仔
Piggy scare ge3 that CL doggy

The doggy that the piggy scared

2. 係 豬仔 嚇 隻 狗仔
Is piggy scare CL doggy

It’s the doggy that the piggy scared

3. 呢 隻 豬 嚇 嘅 狗
This CL pig scare ge3 dog

The dog that this pig scared

4. 公雞 zit1 嘅 兔仔
Chicken tickle ge3 rabbit

The rabbit that the chicken tickled

5. 係 雞仔 zit1 嗰 隻 兔仔
Is chicken tickle that CL rabbit

It’s the rabbit that the chicken tickled

6. 係 雞仔 zit1 嗰 隻 兔仔
Is chicken tickle that CL rabbit

It’s the rabbit that the chicken tickled

7. 貓貓 推 嗰 隻 鴨仔
Cat push that CL duck

The duck that the cat pushed

8. 係 貓仔ong2隻 鴨仔
Is cat push CL duck

It’s the duck that the cat pushed

9. 係 馬仔 踩 嘅 老虎
Is horse step on ge3 tiger

It’s the tiger that the horse stepped on

10. 頭先 隻 馬馬 踩 嗰 隻 老虎
Just-now CL horse step on that CL tiger

The tiger that the horse just stepped on

11. 馬仔 踩 嗰 個 老虎

Horse step on that CL tiger

The tiger that the horse stepped on

12. 係 兔仔 抹 嗰 隻 羊仔
Is rabbit wipe that CL sheep

It’s the sheep that the rabbit wiped

13. 頭先 嗰 隻 獅子 打 嘅 嗰 隻 牛牛
Just-now that CL lion hit ge3 that CL cow

The cow that the lion just hit

14. 係 獅子 嚇 嘅 牛仔
Is lion scare ge3 cow

It’s the cow that the lion scared

15. 係 獅子 踩 嗰 隻 牛仔
Is lion step on that CL cow

It’s the cow that the lion stepped on

16. 頭先 嗰 隻 熊貓 舐 嗰 隻 斑馬
Just-now that CL panda lick that CL zebra

The zebra that the panda just licked

17. 係 熊貓 舐 嗰 隻 斑馬
Is panda lick that CL zebra

It’s the zebra that the panda licked

18. 係 熊貓 舐 嗰 隻 斑馬
Is panda lick that CL zebra

It’s the zebra that the panda licked

19. 貓仔 推 嘅 鴨仔
Cat push ge3 duck

The duck that the cat pushed

20. 馬仔 撞 嘅 老虎

Horse bump ge3 tiger

The tiger that the horse bumped

**Appendix C**

**Illustrative examples of each error type in the SRC condition**

1. **NP only**

Target RC:

錫 (嗰 隻) 雞仔 (嘅 /嗰 隻) 老鼠

Kiss (that CL) chicken (ge3 / that CL ) mouse

The mouse that kissed the chicken

Responses:

呢 隻

This CL

This one

老鼠

Mouse

Mouse

係 呢 隻

Is this CL

It’s this one

1. **Ungrammatical/ irrelevant/ uninterpretable**

Target RC:

錫 (嗰 隻) 雞仔 (嘅 /嗰 隻) 老鼠

Kiss (that CL) chicken (ge3 / that CL ) mouse

The mouse that kissed the chicken

Response:

老鼠 zit1 既 嗰 隻 老鼠

Mouse tickle ge3 that CL mouse

The mouse that the mouse tickled

Target RC:

餵 (嗰 隻) 豬仔 (嘅 /嗰 隻) 馬騮

Feed (that CL) piggy (ge3/ that CL ) monkey

The monkey that fed the piggy

Response:

馬騮 羊咩咩

Monkey Sheep

Monkey Sheep

Target RC:

咬 (嗰 隻) 馬仔 (嘅 /嗰 隻) 熊貓

Bite (that CL) horse (ge3 / that CL ) panda

The panda that bit the horse

Response:

熊貓 大 個 嘅

Panda big CL ge3

Target RC:

咬 (嗰 隻) 馬仔 (嘅 /嗰 隻) 熊貓

Bite (that CL) horse (ge3 / that CL ) panda

The panda that bit the horse

Response:

係 熊人 打

Is Bear hit

It’s the bear hit

1. **(It’s) SVO**

Target RC:

錫 (嗰 隻) 雞仔 (嘅 /嗰 隻) 老鼠

Kiss (that CL) chicken (ge3 / that CL ) mouse

The mouse that kissed the chicken

Response:

老鼠 踢到 雞仔

Mouse kick chicken

The mouse kicked the chicken

呢 隻 老鼠 zit1 呢 個

This CL Mouse tickle this CL

This mouse tickled this one

頭先 呢 老鼠 呢 就 錫 雞仔

Just-now PRT Mouse PRT kiss chicken

The mouse just kissed the chicken

Target RC:

Zit1 (嗰 隻) 羊仔 (嘅 /嗰 隻) 豬仔

Tickle (that CL) sheep (ge3 / that CL ) piggy

The piggy that tickled the sheep

Response:

係 羊仔 zit1 豬仔

Is sheep tickle pig

It’s the sheep tickled the pig

1. **SV**

Target RC:

追 (嗰 隻) 牛牛 (嘅 /嗰 隻) 獅子

Chase (that CL) cow (ge3 / that CL ) lion

The lion that chased the cow

Response:

獅子 追 追 追

Lion chase chase chase

The lion chased

1. **Conversion error to ORC**

Target RC:

錫 (嗰 隻) 雞仔 (嘅 /嗰 隻) 老鼠

Kiss (that CL) chicken (ge3 / that CL ) mouse

The mouse that kissed the chicken

Response:

係 老鼠 追 嗰 個 雞仔

Is mouse chase that CL chicken

It’s the chicken that the mouse chased

1. **VO**

Target RC:

錫 (嗰 隻) 雞仔 (嘅 /嗰 隻) 老鼠

Kiss (that CL) chicken (ge3 / that CL ) mouse

The mouse that kissed the chicken

Response:

頭先 zit1 佢

Just-now tickle it

Just tickle it

1. **SRC with resumptive NP**

Target RC:

撞 (嗰 隻) 斑馬 (嘅 /嗰 隻) 大象

Bump (that CL) zebra (ge3 / that CL ) elephant

The elephant that bumped the zebra

Response:

頭先 嗰 隻 大笨象 撞 嗰 隻 斑馬 既 嗰 隻 大笨象

Just-now that CL elephant bump that CL zebra ge3 that CL elephant

The elephant that the elephant just bumped the zebra

1. **Serial verb construction**

Target RC:

錫 (嗰 隻) 雞仔 (嘅 /嗰 隻) 老鼠

Kiss (that CL) chicken (ge3 / that CL ) mouse

The mouse that kissed the chicken

Response:

老鼠 餵 雞仔 食 野

Mouse feed chicken eat something

The mouse fed the chicken to eat something

**Appendix D**

**Illustrative examples of each error type in the ORC condition**

1. **NP only**

Target RC:

(嗰 隻) 兔仔 抹 (嘅 / 嗰 隻) 羊仔

(that CL) rabbit wipe (ge3 / that CL) sheep

The sheep that the rabbit wiped

Responses:

羊咩咩

Sheep

Sheep

呢 隻

This CL

This one

嗰 隻 嘢

That CL thing

That thing

1. **Ungrammatical/ irrelevant/ uninterpretable**

Target RC:

(嗰 隻) 兔仔 抹 (嘅 / 嗰 隻) 羊仔

(that CL) rabbit wipe (ge3/ that CL) sheep

The sheep that the rabbit wiped

Response:

羊仔 兔仔

Sheep Rabbit

Sheep Rabbit

Target RC:

(嗰 隻) 獅子 打 (嘅 / 嗰 隻) 牛牛

(That CL) lion hit (ge3/ that CL) cow

The cow that the lion hit

Response: (PERF: perfective aspect marker)

佢 做 咗 打 嗰 個

It do PERF hit that CL

Target RC:

(嗰 隻) 熊貓 舐 (嘅 / 嗰 隻) 斑馬

(That CL) panda lick (ge3 / that CL) zebra

The zebra that the panda licked

Response:

熊貓 舐 嗰 長

Panda lick that long

Target RC:

(嗰 隻) 雞仔 zit1 (嘅 / 嗰 隻) 白兔

(That CL) chicken tickle (ge3 / that CL) rabbit

The rabbit that the chicken tickled

Response:

兔仔 雞 餵 佢

Rabbit chicken feed it

1. **(It’s) SVO**

Target RC:

(嗰 隻) 兔仔 抹 (嘅 / 嗰 隻) 羊仔

(That CL) rabbit wipe (ge3 / that CL) sheep

The sheep that the rabbit wiped

Response:

小 白兔 抺 羊仔

Little rabbit wipe sheep

The little rabbit wiped the sheep

Target RC:

(嗰 隻) 豬仔 嚇 (嘅 / 嗰 隻) 狗仔

(That CL) piggy scare (ge3 / that CL) doggy

The doggy that the piggy scared

Response:

係 豬仔 嚇 狗仔

Is piggy scare doggy

It’s the piggy scared the dog

1. **SV**

Target RC:

(嗰 隻) 兔仔 抹 (嘅 / 嗰 隻) 羊仔

(That CL) rabbit wipe (ge3 / that CL) sheep

The sheep that the rabbit wiped

Response:

羊仔 抺 抹 抹

Sheep wipe wipe wipe

The sheep wiped

1. **SVO and with agent-patient role reversal error**

Target RC:

(嗰 隻) 馬仔 踩 (嘅 / 嗰 隻) 老虎

(That CL) horse step on (ge3 / that CL) tiger

The tiger that the horse stepped on

Response:

係 老虎 咬 隻 馬仔

Is tiger bite CL horse

It’s the tiger bit the horse

1. **VO**

Target RC:

(嗰 隻) 熊貓 舐 (嘅 / 嗰 隻) 斑馬

(That CL) panda lick (ge3/ that CL) zebra

The zebra that the panda licked

Response:

鍚 斑馬

Kiss zebra

Kiss the zebra

1. **Serial verb construction**

Target RC:

(嗰 隻) 兔仔 抹 (嘅 / 嗰 隻) 羊仔

(That CL) rabbit wipe (ge3 / that CL) sheep

The sheep that the rabbit wiped

Response:

呢 隻 兔仔 幫 佢 抺 野

This CL rabbit help it wipe stuff

This rabbit helped it to wipe something

1. **ORC with resumptive pronoun**

Target RC:

(嗰 隻) 豬仔 嚇 (嘅 / 嗰 隻) 狗仔

(That CL) piggy scare (ge3 / that CL) doggy

The doggy that the piggy scared

Response:

頭先 嗰 隻 豬仔 嚇 佢 嗰 隻 狗狗

Just-now that CL piggy scare it that CL doggy

The doggy that the piggy just scared it.
